# Supplementary material for: MetaRibo-Seq measures translation in microbiomes
Source: Nat Commun. 2020 Jun 29;11:3268. doi: 10.1038/s41467-020-17081-z (PMC7324362; doi:10.1038/s41467-020-17081-z)
Supplement: Supplementary file 10 — Supplementary Data 7 [file 41467_2020_17081_MOESM10_ESM.zip › File2/Confidence_High_Taxonomy/97143_out.krona.html]

Javascript must be enabled to view this page.

members
magnitude
magnitudeUnassigned
count
unassigned
taxon
rank

97143\_out

10

10
superkingdom
2

10
976
phylum

class
200643
10

order
171549
10

815
family
10

10
genus
816

SRS077231\_contig\_number\_22790
1

1
457389
species

SRS050026\_contig\_number\_11957


SRS143085\_contig\_number\_6246SRS148970\_contig\_number\_contig-100\_655.36441
2
817
species

6
457390
species

SRS013940\_contig\_number\_contig-100\_162.111910SRS046068\_contig\_number\_5061SRS062701\_contig\_number\_contig-100\_1138.62508SRS1041142\_contig\_number\_6304SRS1055038\_contig\_number\_1059SRS148091\_contig\_number\_10912
